# A Dissociation of Attention, Executive Function and Reaction to Difficulty: Development of the MindPulse Test, a Novel Digital Neuropsychological Test for Precise Quantification of Perceptual-Motor Decision-Making Processes

**DOI:** 10.3389/fnins.2021.650219

**Published:** 2021-07-19

**Authors:** Sandra Suarez, Bertrand Eynard, Sylvie Granon

**Affiliations:** ^1^It’s Brain SAS, Orsay, France; ^2^IHES, Institut des Hautes Études Scientifiques, Bures-sur-Yvette, France; ^3^IPHT/DRF/CEA Institut de Physique Théorique, Gif-sur-Yvette, France; ^4^Université Paris-Saclay, CNRS, Institut des Neurosciences Paris-Saclay, Gif-sur-Yvette, France

**Keywords:** digital test, attention, executive functions, decision making, software, psychomotor speed, reaction time, categorization

## Abstract

Traditionally, neuropsychological testing has assessed processing speed and precision, closely related to the ability to perform high-order cognitive tasks. An individual making a decision under time pressure must constantly rebalance its speed to action in order to account for possible errors. A deficit in processing speed appears to be afrequent disorder caused by cerebral damage — but it can be hard to pinpoint the exact cause of the slowdown. It is therefore important to separate the perceptual-motor component of processing speed from the decision-time component. We present a technique to isolate Reaction Times (RTs): a short digital test to assess the decision-making abilities of individuals by gauging their ability to balance between speed and precision. Our hypothesis is that some subjects willaccelerate, and others slow down in the face of the difficulty. This pilot study, conducted on 83 neurotypical adult volunteers, used images stimuli. The test was designed to measure RTs and correctness. After learning release gesture, the subjects were presented with three tasks: a simple Reaction Time task, a Go/No-Go, and a complex Go/No-Go with 2 simultaneous Choices. All three tasks have in common a perceptual component and a motor response. By measuring the 3 reference points requiring attentional and executive processing, while progressively increasing the conceptual complexity of the task, we were able to compare the processing times for different tasks — thus calculating the deceleration specific to the reaction time linked to difficulty. We defined the difficulty coefficient of a task as being the ratio of the group average time of this task minus the base time/average time of the unit task minus the base time. We found that RTs can be broken down into three elementary, uncorrelated components: Reaction Time, Executive Speed, and Reaction to Difficulty (RD). We hypothesized that RD reflects how the subject reacts to difficulty by accelerating (RD < 0) or decelerating (RD > 0). Thus we provide here a first proof of concept: the ability to measure four axes of the speed-precision trade-off inherent in a subject’s fundamental decision making: perceptual-motor speed, executive speed, subject accuracy, and reaction to difficulty.

## Introduction

The goal of a clinical test is to observe a difference between a normal condition and a pathological condition. To achieve this, it is necessary to measure certain parameters, among which the precision of the subject’s response (number and quality of errors) and processing speed. Processing speed is measured using Reaction Times (RTs). The reaction time to the onset of a visual stimulus is a well-studied behavioral measure that presents moment-to-moment fluctuations (Ribeiro et al., 2016). Speed and precision are closely related to the ability to perform higher-order cognitive tasks and encompass many components including perceptual, cognitive and output speed (Kibby et al., 2019).

The reaction time (RT) is interesting in several respects. It is a continuous measurement (without an arbitrary upper limit) and is sensitive to small differences in performance (Teng, 1990). Psychomotor slowing is also thought to be linked to alteration in the subcortical-frontal loops, to a frontal involvement (Kochunov et al., 2010), and to diffuse white matter damage. Psychomotor slowing is a fundamental symptom of neuropathological disorders; for example, in HIV encephalopathy, psychomotor slowing is the only precursor sign of dementia (Sacktor et al., 1996) and is the first symptom to improve under treatment (Stankoff et al., 2001; Suarez et al., 2001), suggesting that it may reflect neuroanatomical damage. Psychomotor speed is also a measure that has been reported to be significantly slower in psychiatric conditions such as schizophrenia (Osborne et al., 2020). Studies focusing on Attention Deficit/Hyperactivity Disorder (ADHD), for instance, have shown that mean response times are significantly higher than those of the control group, the difference being more marked in a subgroup with a more executive profile. It therefore seems necessary to examine these profiles by dissociating the cognitive elements as far as possible (Nigg et al., 2005; Willcutt et al., 2005). Another factor that seems to intervene in psychomotor speed is motor speed. Neuropsychological testing has traditionally assessed processing speed with paper-and-pencil tests, but results may be confounded by motor speed especially in older participants (Ebaid et al., 2017), as reaction times slow down and become more variable with age (Der and Deary, 2006; Ebaid et al., 2017). It should also be taken into account that psychomotor speed is a parameter that varies greatly from one individual to another and that it also varies for the same individual due to circadian variation (Gueugneau et al., 2017). It appears important, therefore, to distinguish the perceptive-motor part of processing speed, particularly with clinical populations in whom deficits in motor performance are frequently observed (i.e., in aging) (Der and Deary, 2006; Ebaid et al., 2017). However, speed is not the only issue. The crucial question behind speed measurement is that a change in speed may reflect an alteration in cognitive functions, in particular decision-making processes (Ernst and Paulus, 2005; Doya, 2008), in other words if there is a “decision time.”

Decision-making is a fundamental adaptive process that allows an individual to choose one option among several (Pittaras et al., 2018; Cabeza et al., 2020), the goal being to choose the most advantageous option in both the short and the medium term. This neurobiological process brings into play several cognitive, affective and motivational functions associated with the activation of brain networks that rely on coordinated brain structures, among which the prefrontal cortex, amygdala, insula, and nucleus accumbens play critical roles (Volz et al., 2006; Pittaras et al., 2016). It also depends on the context in which the individuals find themselves and on their own needs and internal feelings (Bechara et al., 2000). The influence of the individual’s affective state on decision making has been explored in particular by the theory of somatic markers (Bechara et al., 1994) which suggested that, under uncertain circumstances, second-level processing of the intact emotion system could facilitate rational decision making in the long term (Damasio, 1994, 1996). Somatic markers and the environment influence the speed of decision-making processes; it is therefore the combination of the individual’s emotional, motivational and cognitive state as well as the characteristics of the environment that will allow the person to choose one option over another more rapidly (Ernst and Paulus, 2005; Doya, 2008). These abilities are sensitive to the environment and in particular to lack of sleep (Rabat et al., 2016; Pittaras et al., 2018). Decision making is therefore the result of a balance in which different processes intervene. This balance constantly adjusts a system to take errors into consideration. In sensorimotor activities performed under time pressure, the action monitoring system acts before, during, and after the action in order to take errors into account (Vidal et al., 2020). Hence, attentional processes underlie decision-making processes: being engaged in a cognitive task triggers a general maintenance of vigilance/alertness in order to achieve the fastest reaction time possible (Godefroy et al., 1999), and stimulus presentation regularly attracts attention. Processing speed is ecologically essential for adaptation to our environment. A deficit in this function appears to be one of the most frequent attentional disorders caused by cerebral damage (Godefroy et al., 2018). It is important, therefore, to understand the mechanism of slowness and in particular, to understand whether cognitive slowing is linked to motor slowing. Therefore, one way to observe decision making is to measure the balance between time and response accuracy and, as discussed above, since speed is broken down into motor speed and cognitive speed, it would no doubt be informative to explore decision making from a clinical perspective in order to break down its component elements of speed and precision.

Executive functions (EFs) refer to the top-down mental processes needed when someone has to concentrate and pay attention. There are three core EFs: inhibition (involving being able to control one’s attention), working memory, and cognitive flexibility (Diamond, 2013; Friedman and Miyake, 2017). Since the work by Crick (1984) in the mid-1980s, attention has been described as a kind of spotlight that can be controlled. Subsequently, Posner and Petersen (1990) developed the theory of three networks of attention systems: an alerting network, related to sustained vigilance, an orienting network, and an executive network. Updating the model in 2012, Petersen and Posner introduced the notion of “self-control,” namely the ability to control our thoughts, feelings, and behavior, revealed in conflict tasks such as the Stroop task (Petersen and Posner, 2012).

To assess the presence of dysfunction in executive and attentional functions, neuropsychological tests and questionnaires are used to specify the pathological profile of cognitive functions. For example, focusing on ADHD, Sonuga-Barke (2002) proposed a model which posits the existence of multiple neurodevelopmental pathways, suggested by the existence of patient subgroups showing different profiles of neuropsychological dysfunctions. Most of these tests measure the speed of the subject’s reaction and his/her accuracy, and many experiments have proved that objects that better capture attention are processed faster and with higher accuracy, and that allocating attention to the target location speeds up the reaction in probe detection tasks (Posner, 1980). Drawing on these findings that attention enhances processing, models to measure visual processing speed and the distribution of attentional resources have been proposed (Tünnermann et al., 2017). As a result, most neuropsychological clinical tests require the subject to accomplish a task as fast as possible, or to perform as many actions as possible in a limited amount of time. However, tests are constrained by the duration of the task that is being observed. It has been found that if a reaction time task exceeds 2′30′′, then what is measured is not selective attention but the effect of fatigue, and the intra individual variability in response time increases with time-on-task (Tarantino et al., 2013). Consequently, the duration of the test or the subtest is important depending on what we are trying to measure, and if we want to measure selective attention, the duration must be short (i.e., around 2′30′′). The neuropsychological tests widely used to measure these functions, (because they are sensitive to a variety of neurological disorders) such as the STROOP (Stroop, 1935) and the Trail Making Test (Reitan, 1992), focus on measurements within the first 2 min, and use very few data (2–3 speed data and 2–3 precision data). They therefore avoid conditions of fatigue and yet manage with small amounts of data to distinguish a normal condition from a pathological condition.

Among the factors that may influence RT, Sternberg (1966) assumed that there must be an effect of task complexity on processing speed. In the diffusion model, a prominent cognitive process model of speeded two-choice decisions (Ratcliff and McKoon, 2008), one of the main parameters, the drift rate, is claimed to be related to the task difficulty and to individual differences in the quality of information processing. It is therefore reasonable to think that psychomotor slowing could be measured in a decision-making task by making the choice more difficult or complex. We will examine two ways of breaking down RTs: the Godefroy process (Godefroy et al., 2010), and the diffusion model (Ratcliff and McKoon, 2008). The former proposes that in a simple reaction time task, four critical processes are involved: (i) a perceptual process that determines the relevance of the stimulus; (ii) a decision process that triggers a behavior (the motor response); (iii) an action process; (iv) a central attentional process. With the repeated reaction time curve observation model, Godefroy et al. (2010) observed that slowing was mainly linked to perceptual and motor processes and that it was only after the age of 60 that attentional markers played a significant role. In this descriptive model of RTs, the “perceptual and motor” part is not independent of the others. The idea of removing the perceptual-motor part by a subtraction is in fact contained in the diffusion model (Ratcliff and McKoon, 2008). The diffusion model provides a theoretical account of performance in speeded two-choice tasks and has been successfully applied across a wide range of paradigms. Fitting the ex-Gaussian function (for a review, see Ratcliff and McKoon, 2008) to empirical RT data provides estimates of three independent parameters: Mu represents the mean of the normal component and mainly reflects average performance; Sigma corresponds to thestandard deviation of the Gaussian portion of the RT distribution and indicates variability of performance; Tau corresponds to the variability of the exponential function, associated with the skewness of the tail of the RT distribution, and reflects extremes in performance (Matzke and Wagenmakers, 2009).

As RTs are classically used as indices for measuring attentional functions, it seems essential, in view of its variability, to dissociate the various components of the RTs and separate the perceptuomotor components of reaction times from their attentional and executive components in order to better understand what really generates slowing in pathologies. By breaking RTs down into its different components, it should be possible to highlight the underlying elements of perceptuomotor decision-making, particularly attentional and executive functions.

In order to detect and characterize attentional and executive deficits and reaction to difficulty in mild cognitive impairments in the scope of the perceptivo-motor decisional abilities, we propose a new neurocognitive digital test named “MindPulse.” The index used in our test reflects precision and psychomotor slowing, determined by measuring RTs and errors in various conditions of difficulty. In order to avoid cultural biases, the test was developed from knowledge gained from animal cognition. Many studies have shown categorical responses in different species such as pigeons (Wright and Cumming, 1971), goldfish (Poralla and Neumeyer, 2006) and macaques (Walsh et al., 1992). The conceptualization capacity of primates was explored by Fabre-Thorpe (2003) in a go/no-go categorization task using colored versus gray level photographs and with abstract concepts such as ‘food objects’ or ‘animals.’ The earlier study by Thorpe et al. (1996) was the first to give a direct estimate of the processing time necessary to perform fast visual categorizations. In the study, primates had to categorize stimuli based on whether they were animals or not. It was found that their RTs were around 300 ms. When the same task was conducted in humans, it was found that RTs fell to less than 150 ms (Thorpe et al., 1996). The authors also provided a precise description of the characteristics and temporal evolution of visual categorization in the primate brain and suggested that the processing of visual information during the activation of abstract representations was analogous in humans and monkeys (Fabre-Thorpe, 2011; Fize et al., 2011). Taking into account the existence of these similarities, we used general categorizations to primates (e.g., white versus gray, and animals versus object).

As mentioned above, RTs include not just visual processing but also the decision process and the motor time. The minimum time required to generate a “reaching command” in humans is 80–100 ms (Kalaska and Crammond, 1992) and the necessary visual processing mechanisms involved in the categorization tasks take about 150 ms (Thorpe et al., 1996; VanRullen and Thorpe, 2001). Teng (1990) introduced the idea of separating the cognitive and the sensorimotor processes and subtracting the latter from the total reaction time. However, the mathematical modeling of the levels of complexity was not exploited. In the Trail Making Test, there are two parts: part A consists in connecting numbers in ascending order and part B consists in alternating numbers and letters in numerical and alphabetical order. The B/A ratio provides an index of executive function (Arbuthnott and Frank, 2000). However, when calculating the ratio, the motor and the executive steps are considered to be proportional.

Here, our aim was to break down fundamental decision-making processes into their attentional, executive and maybe psychological components. We present a technique to isolate Reaction Times (RTs): a short digital test to assess the decision-making abilities of individuals by gauging their ability to adapt — how they strike the balance between speed and precision — relative to their perception of the complexity of the given task. Their capacities of adaptation should lead some subjects to go faster, while others slow down in the face of the difficulty. We hypothesize that by superimposing three tasks using the same visual presentation and the same motor response (varying only the difficulty of the categorization to be carried out), we will be able to break down the reaction time into more basic and independent parts.

The projected output of the test was to provide an easy and fast way to measure these different brain processes in any individual regardless of his/her culture, language or reading ability.

In addition, the test requires the subject’s commitment to action in order to control for motivational aspects. We used a procedure mimicking that used in experimental psychology in rodents (Brasted et al., 1998). In their study, the authors developed a procedure to ensure the animal’s attention by training the rat to sustain a nose poke for a variable period of time until a brief unpredictable visual stimulus appeared in one of the two side holes. Reaction time was then defined as the latency to withdraw the nose from the center hole of a Skinner box after onset of the light stimulus, and movement time was defined by the latency to poke its nose through another hole. Here, we used a similar procedure adapted to humans in order to ensure attentional commitment of the subjects by asking them to keep their finger pressed on the mouse until a stimulus of the appropriate category appeared on the screen (see “Materials and Methods” section).

## Materials and Methods

### Underlying Principles of Test Development

The MindPulse test is an original digital software protected by a patent (Suarez et al., 2019). In this test, the subject is seated in front of a computer screen that shows images to which he/she is instructed to respond via a wired mice mouse. The test consists of four parts of increasing complexity (see section “Description of the Test” below) that the subject has first to learn (learning phase) and then to perform (test phase). The test starts with subjects first learning a “release action,” i.e., the subject clicks on the mouse, maintains the pressure on the mouse button until a stimulus is shown on the screen, and then releases his/her finger. This procedure, borrowed from animal experiments, ensures the subject’s engagement.

**FIGURE 1:**
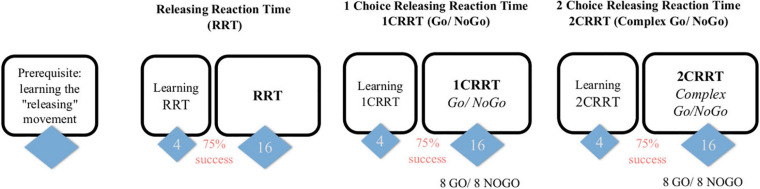
Subjects first learn the releasing gesture (subject is in front of the screen and responds with the mouse, the response is by releasing the mouse rather than clicking) and then have 3 tasks, of increasing difficulty, each one preceded by a learning part (4 trials and 3/4 must be passed to proceed to the test part). The first task consists in a “simple Releasing Reaction Task” (RRT) with 16 trials. The second is a one-choice Releasing Reaction Task (1CRRT) with 8 Go and 8 NoGo. The third is a two-simultaneous Choices Releasing Reaction Task (2CRRT) with 8 Go and 8 NoGo.

Stimuli are single images displayed in the center of the screen on a white background. The images were designed to be recognized and classified without cultural bias (see below). In order to avoid effects due to stimulus repetition, the task uses a wide variety of pictures that are seen only once in each part. The strict identity in the perceptual part and the motor response in all the tasks of the test is an essential principle of the MindPulse test. Only the instructions and the rules to be applied are different. An overview of the MindPulse timeline is presented in Figure 1. The computer records reaction times and the quality of responses (number and type of errors) for each trial.

### Patient Information Requested

This pilot study was conducted as a “proof of concept” and was not devoted to establishing standards. We recruited 83 neurotypical adult volunteers [19–45 years old, 63 women, 72 right-handed]. All the subjects declared that they had no neuro-cognitive, neurological, or psychiatric medical history or neuro-cognitive damage, no neuroactive treatment or drug intake in the past 3 months, including sleeping medication, antidepressants and anxiolytics, and no alcohol addiction. In addition, they specified their native language, whether they were right- or left-handed, and completed a questionnaire on their current psychological state, the Hospital Anxiety and Depression scale (HAD) (Zigmond and Snaith, 1983).

The subject’s name and birth date (month/year) were not recorded but were typed in a password-like entry form on the computer and instantly hashed into an anonymized unique identification code. The goal of this code was to match data for subjects who did a retest a few days later.

### Procedure

The experiment was conducted in a classroom at Paris-Saclay University. Subjects were seated in front of the computer at a distance of about 60 cm from the screen and were encouraged to use their preferred hand. Experimenters ensured that the instructions had been well understood. Three computers with a wired mouse and with the MindPulse test software installed were used in the experiment. To explore the retest effect, a subgroup of 28 subjects were retested the following day.

### Data Collection

**FIGURE 2:**
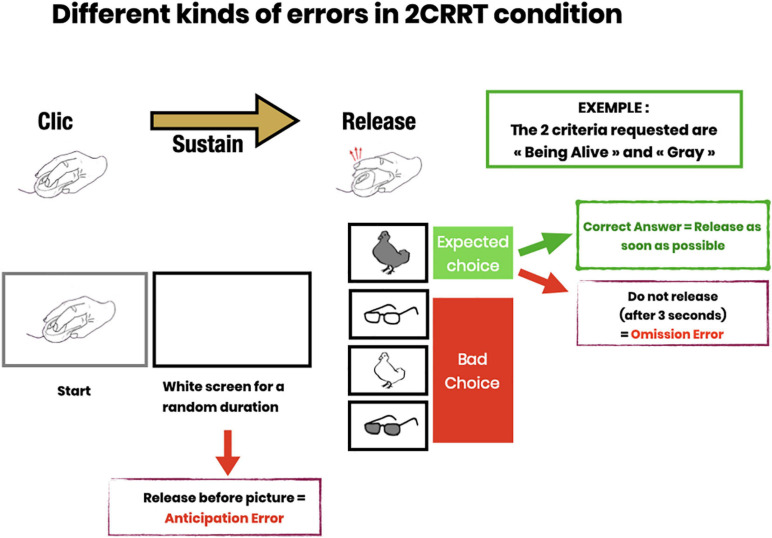
Description of error subtypes. Anticipatory errors (subject releases pressure on the mouse button before the image appears); Omission errors (subject does not release within 3 s after image presentation); Choice errors (subject responds to the wrong category).

The test measures the reaction time equal to the latency in clicking or releasing the mouse button after the image appears. The precision is about 3 hundredths of a second [limitation set by the screen refreshment time of 16 ms, by the mouse (about 10 ms for a wired mouse), and the OS: the time recording software programmed by a professional software engineer is in C-language, a compiled low-level language, usually taking nanoseconds to execute]. The test also records for each stimulus the white screen duration (time before the image appears drawn by a semi-random constrained algorithm) and measures the correctness (right or wrong) and type of response (not releasing or releasing before or after the image appears) (See Figure 2).

### Description of the Test

The test has four main parts (see Figure 1). In each part the subject has to respond to an image appearing on the screen:

• Preliminary: learning the “releasing movement.”
• Simple Releasing Reaction Time Task (RRT): the subject has to respond to any image.
• A Go/No-Go task with 1 Choice Releasing Reaction Time (1CRRT): the subject has to respond only to images of a prescribed color category, white or gray.
• And a complex Go/No-Go task with 2 simultaneous Choices Releasing Reaction Time (2CRRT): the subject has to respond only to images of two prescribed categories, i.e., color white or gray, and nature alive (animate) or not-alive (inanimate).

In each task, the subject received instructions on the screen, then performed the trials. In each trial, an image instructing the subject to press the mouse appeared, then as soon as the mouse button had been pressed, the screen turned blank for a random amount of time (between 2 and 7 s called “waiting time”) during which the subject had to maintain the pressure on the mouse. After the waiting time, an image appeared, and the subject had to release the mouse as fast as possible if the image satisfied the go/no-go criteria. If the image did not satisfy the criteria, the subject was asked to release the button after 3 s.

The 1-choice go/no-go category criterion was chosen at random between color = white or color = gray, at the beginning of the test. The 2-choice go/no-go category criterion was set as the opposite color of the 1-choice task, plus an additional category chosen at random between nature = animate or nature = inanimate.

The images used in the test were chosen at random from an image bank specially created for the test and carefully selected so that any healthy individual in any country would be able to classify them according to the animate/inanimate criterion (i.e., animals and objects were chosen to be without cultural bias). The random algorithm ensured that no image could appear twice (independently of its color) during a task. Two distinct image banks were used for the learning and test phases.

The random waiting times before each image were selected by a constrained random algorithm to ensure that waiting times were in the range of 2–7 s, and well distributed among the color and nature categories.

In the simple RRT task, the subject had to release the mouse after the appearance of any image, whereas in the go/no-go tasks the pressure on the mouse had to be released only if the image satisfied 1 or 2 criteria.

Each of the three tasks, RRT, 1CRRT, and 2CRRT, was preceded by a learning period with four trials. If the subject made an error, the experimenter intervened to explain it. Subjects who failed more than once out of the four training trials were asked to repeat the four training trials once or twice. Subjects who failed more than three series of four training sessions were to be excluded. This never occurred in the current pilot study. Subjects then accessed the test parts after successfully completing the training session.

In each trial, the subject started by pressing the button of the mouse on seeing the “clicking hand” image. Immediately after clicking, the signal disappeared, and the screen turned white. The latency before the appearance of the target image was set according to a semi-random algorithm, between 2 and 7 s. After the target image appeared, the subject had 3 s to respond. Each part of the task consisted of 16 successive trials. No information was provided to the subjects as to whether they passed or failed during this test phase. In the go/no-go tasks, 8 images corresponded to the target, and 8 images did not. Therefore in the absence of errors, there were 16 reaction times measured in the first task, 8 in the simple go/no-go task and 8 in the 2-choice go/no-go task, amounting to 32 reaction time measures altogether. Errors were rare (90% of subjects made at most 1 error). The presence of errors could diminish the precision of the time measurement, but the number of errors was considered to be inherently more useful information about pathological conditions than the precision of reaction time.

#### The Releasing Movement: AKey to Attentional Engagement

The first task involved learning the gesture of clicking on the mouse button at the signal and releasing it when an image appeared. A “clicking hand” picture prompted the subject to click on the computer mouse (see Figure 2). The experimenter explained the instruction to the subject: “Keep your finger pressed as long as the screen remains blank. Then release the mouse button as quickly as possible when an image appears.” The subject was invited to practice as many times as necessary.

#### The Releasing Reaction Time Task (RRT)

The Releasing Reaction Time Task (RRT) is a measurement of the subject’s releasing reaction time. The subject has to disengage the motor gesture when the target image appears. This task includes a learning part, (4 trials and 3/4 had to be passed to proceed to the test part) then a test part with 16 trials (see Figure 2). Behavioral measures recorded Reaction Time; Time before the target image appears; Anticipation Errors; and Omission Errors.

#### The 1CRRT (1-Choice Releasing Reaction Time) Is a Go/NoGo Releasing Task

1CRRT provides a measurement of the subject’s reaction time with one choice, and errors in the form of a Go/No-Go task. The category is a color choice (the subject must release the mouse button if the image is gray or white). The target category was randomized between subjects.

Subjects were instructed to release the mouse button (Go response) as quickly as possible when the picture was the target. They had 3 s to perform a Go response after which any response was considered as a no-go response. This task included a learning part, (4 trials and 3/4 had to be passed to proceed to the test part) then a test part with 16 trials, 8 Go and 8 NoGo (see Figure 2). The behavioral measures recorded were: Reaction Time; Time before the target image appears; Anticipation Error; Omission Error; Right or Wrong choice (button released when the image was not the target category).

#### 2CRRT (2-Choice Releasing Reaction Time) Is a Complex Go/No-Go Releasing Task

In the 2CRRT task, the subject had to react (release) only for stimuli corresponding simultaneously to the two required categories. The color criterion was systematically reversed with respect to the 1CRRT, which requires inhibiting the previously relevant color. The second, new criterion corresponds to the animate/inanimate nature of the picture stimuli. The selection of the relevant criterion for this new category was random. The 2CRRT included a learning part (4 trials and 3/4 had to be passed to proceed to the test part), while the 2CRRT Test part comprised 16 trials, 8 Go and 8 NoGo (see Figure 2). The behavioral measures scored were: Reaction Time; Time before the target image appears; Anticipation Error; Omission Error; Right or Wrong choice and details about the kind of wrong choice errors (Figure 2).

### Code

The test used to collect data was a research prototype (named TIREX) developed in Python by a professional software engineer, running on a MacbookPro computer. The prototype was usable exclusively on the researchers’ computers and was not subjected to an operating license. The data were then processed using the Python, *scipy.stats, sklearn, statsmodels, matplotlib, seaborn*, and *Pandas* libraries, which are standard libraries in data science.

A patent of the test has been filed (Suarez et al., 2019). The prototype was developed for a dedicated computer, and sources are available for reviewers. The authors cannot be held responsible for environment compatibility on another computer than the one for which it was developed.

The TIREX prototype was not developed for scalability, but clinicians can have access to the upgraded version (subject to license and patent) which performs identical measurement tasks, with an improved design, ergonomics, precision, portability to other systems, and user interface, making the learning phase autonomous (not requiring the researcher’s explanations and supervision). The upgraded version was named MindPulse.

## Data Analysis Program

The test produces 16 + 8 + 8 reaction times per subject, plus other data amounting to a total of 144 data per subject. Each subject accomplishes 3 × 16 tasks, for which we recorded 3 items: the reaction time, the correctness of the response, and the type of response (release or no release, after or before the image appears).

We first extracted the list of error-free reaction times. We calculated a mean of each individual’s RT. The RT variation index (standard deviation) is very sensitive to extreme values and its reliability is low for individual RT measurements (Brewer and Smith, 1984). Nevertheless, it is an interesting measure which reflects fluctuations in attentional and executive control as well as impairments in information processing and, in particular, a dysfunction related with a failure to maintain attentional control (Haynes et al., 2017). It is greater among children with Attention-Deficit/Hyperactivity Disorder than among typically developing controls. It also appears to be characteristic of other populations, including autism spectrum disorders, schizophrenia, and traumatic brain injury, and has been commonly observed in the elderly (Tamm et al., 2012; Graveson et al., 2016; Haynes et al., 2017). As this is a proof of concept of a test and not of clinical trials, we will only show our measures of variability, the objective then being to carry out standards of these measures in order to be able to observe the variations. in pathological conditions.

### Measures: Methods and Concepts

#### Primary Measurements

Our primary measurements were the following:

RRT: average reaction time over the first task (no choice), calculated only on valid responses (maximum 16). 1CRRT: average reaction time over the second task (1-choice go/no-go), calculated only on valid responses (maximum 8). 2CRRT: average reaction time over the third task (2-choice go/no-go), calculated only on valid responses (maximum 8). Having less than maximum number of response times is rare in normal subjects. Moreover in view of developing a clinical test, evaluating a balance between rapidity and precision, we consider that subjects with several errors will be evaluated in a balance between the number of errors and rapidity.

#### The Difficulty Scale

Our aim was to extract information about decision time from the participant data. The idea was to first subtract for each subject the mean RRT from the choice RRTs, assuming that the subtraction gives an executive component of the response, and then compare the reaction times for tasks of different levels of difficulty, by establishing a “difficulty scale.”

We considered that the RRT plays the role of a baseline (perceptivo-motor part of the response) and the remaining part is interpreted as the executive part, and is proportional to the difficulty of the task. This construct is in agreement with fundamental neuroscience theory of reaction time. In particular in the diffusion model (Ratcliff and McKoon, 2008), one parameter is a baseline (the non-decisional time) and another one is the drift (time scale related to the difficulty of the task), and in this theoretical model, after subtracting the baseline and keeping all parameters equal, the time distributions are proportional to the inverse of the drift parameter). Therefore, we proceeded as follows:

We defined the ChoiceReactionTimes with baseline subtracted (averages for a subject):

$$1CRRT^{\prime }=1CRRT-\mathrm{RRT}$$

$$2CRRT^{\prime }=2CRRT-\mathrm{RRT}$$

The statistical distributions of 1CRRT′ and 2CRRT′ were of course different with different averages and standard deviations. We then multiplicatively rescaled the 2CRRT′ by a coefficient which brings its group average to the same value as that of 1CRRT′. We defined Coef as the ratio of group averages of 2CRRT′ by 1CRRT′:

$$Coef=groupaverage\left(2CRRT^{\prime }\right)/groupaverage\left(1CRRT\right)$$

**FIGURE 3:**
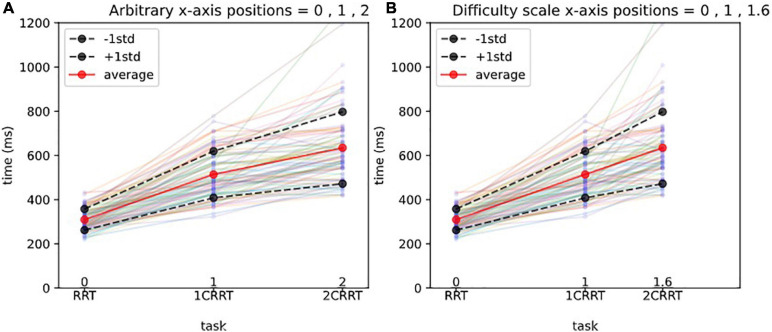
Difficulty scale for reaction times.**(A)** Arbitrary *x*-axis positions. The *x*-axis represents the 3 tasks, and the *y*-axis the reaction times (in ms). Each line with 3 points represents the reaction times of a subject, and the thick lines represent the average and 1std lines of the 83 subjects. Without a notion of difficulty scale, one could represent arbitrarily the 3 tasks at positions 1,2,3 on the *x*-axis, as shown in **(A)**.**(B)** Difficulty scale *x*-axis positions. In **(B)** we represent the 3 tasks, respectively, at positions 0, 1, 1.592, which makes the averages aligned. In other words, we consider that the 0 Choice task has difficulty 0, the 1 Choice task serves as a unit and is at position 1, and the 2 Choices task is 1.592 times more difficult than the 1 Choice task. The average values of 2CRRT′′ and 1CRRT′ become equal.

The value found in our subject data was Coef = 1.59 (95% confidence interval = [1.29, 1.76). We named this coefficient the “Difficulty Coefficient.” It introduces a notion of difficulty scale (Figures 3A,B).

We then defined 2CRRT″ = 2CRRT′/Coef, as a rescaled 2-Choice-executive time on the same scale as that of the 1-Choice 1CRRT′.

$$2CRRT^{\prime \prime }=2CRRT^{\prime }/Coef=\left(2CRRT-RRT\right)/Coef$$

**FIGURE 4:**
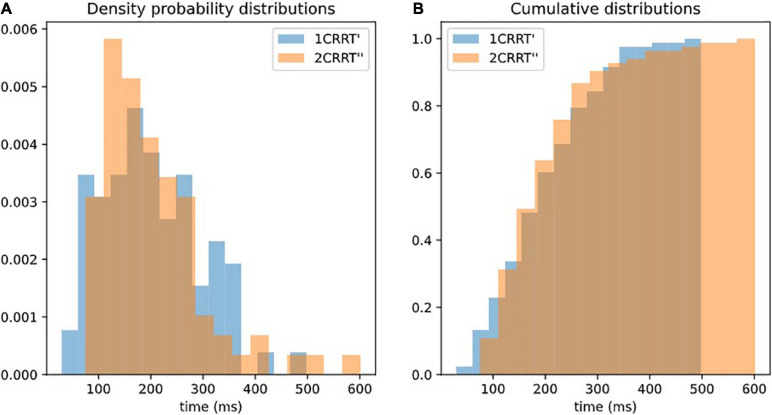
Superposition of distributions of 1CRRT′ and 2CRRT′. After re-scaling 2CRRT for difficulty, the 2 distributions 1CRRT′ and 2CRRT′are superposed. KS, ***d*** = 012, ***p*** = 0.59. The standard deviations also become equal and the whole distribution over the 83 subjects is the same (the Kolmogorov–Smirnov test gave ***d*** = 0.12, ***p*** = 0.59 against the hypothesis of different distributions). Our findings can be interpreted as a confirmation that the difficulty corresponds to a scaling parameter. **(A)** Distribution of 1CRRT′ and 2CRRT′′ are superposed. **(B)** View of the cumulative distribution of 1CRRT′ and 2CRRT′′.

Our hypothesis was confirmed by the fact that not only the average values of 2CRRT″ and 1CRRT′ become equal (Figure 3B) but the standard deviations also become equal and the whole distribution over the 83 subjects is the same [the Kolmogorov–Smirnov test gave *d* = 0.12, *p* = 0.59 against the hypothesis of different distributions (Figure 4)]. Our findings can be interpreted as a confirmation of the agreement with the diffusion model hypothesis that the difficulty corresponds to a scaling parameter.

#### Secondary Measures

From the primary measures and using this difficulty coefficient, we defined secondary measures:

We defined the “Executive Speed” (ES): average executive time was obtained by subtracting the 0-Choice RRT from both the 1-Choice RRT (1CRRT) and the 2-Choice RRT (2CRRT) in order to subtract the perceptual-motor part, and then rescaling 2CRRT′ using the formula:

$$ES=\left(1CRRT^{\prime }+2CRRT^{\prime \prime }\right)/2$$

**FIGURE 5:**
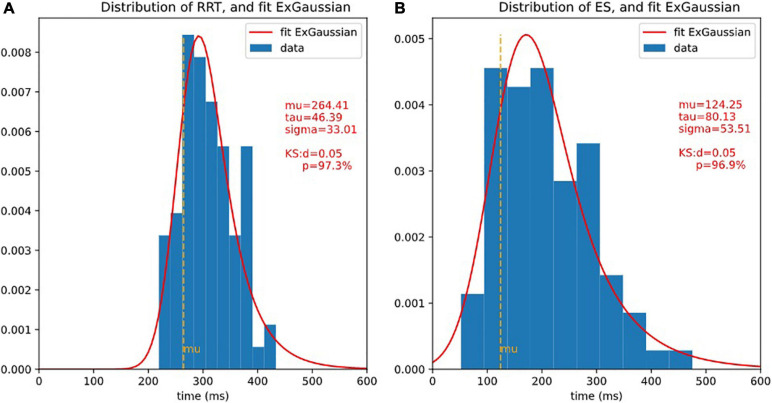
Distributions of RRT and ET and ex-Gaussian fit. It is usual to fit reaction times with ex-Gaussian distribution. 3 ex-Gaussian parameters are considered: mu is the location (close to the center), sigma represents the width, and tau represents the length of the exponential tail. The quality of the fit is assessed by Kolmogorov-Smirnov test (KS), the smaller *d* (higher *p*), the better fit. Here, the fit is extremely good. **(A)** Histogram of RRT and fit ex-Gaussian. **(B)** Histogram of ES and fit ex-Gaussian.

The statistical distribution of ES (see Figure 5 for the distribution) was as expected identical ‘(not statistically distinguishable) from that of 2CRRT″ or 1CRRT′ (KStest ES/1CRRT′: *d* = 0.096, *p* = 0.83, KStest ES/2CRRT″: *d* = 0.108, *p* = 0.71).

We also recorded information about how the subject reacted to difficulty. We defined:

$$\Delta =\left(2CRRT^{\prime \prime }-1CRRT^{\prime }\right)/2$$

In consequence, ES and Δ represent the same information as 1CRRT′ and 2CRRT′′ but rotated by 45°.

Subjects with Δ < 0 accelerated when the difficulty increased (in fact they slowed down less than average), whereas subjects with Δ > 0 slowed down more than average. This measure was therefore a good index of the reaction to difficulty.

**FIGURE 6:**
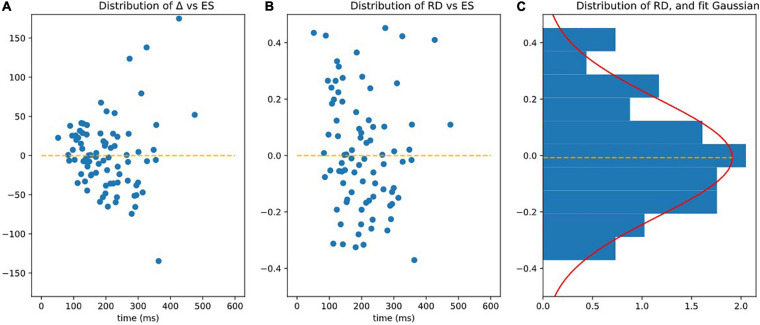
Observing homoscedasticity of RD. **(A)** It is visible on figure that the variance of Delta grows with ES (slope of variance = 24.80, *r*-value = 0.470, *p*-value = 7.36e-06), meaning that Delta is not homoscedatic. **(B)** Represents RD = Delta/ES, which is homoscedatic (assessed by the fact that the square of RD is not correlated to ES significantly *p* > 0.05). **(C)** Is the distribution of RD fitted by Gaussian.

Reaction to Difficulty (RD): As such, the residue Δ was not homoscedastic (see Figure 6A). Statistically speaking, it depended on the values of ES, as subjects with a higher ES also had a larger Δ (slope of variance = 24.80, *r*-value = 0.470, *p*-value = 7.36e-06). By dividing by ES we defined the following index

RD=Δ/ES

The RD index was homoscedastic and its standard deviation was independent (slope of variance = 0, *r*-value = 0.068, *p*-value = 0.539 NS) of ES (see Figure 6B).

RD had a centered Gaussian distribution (σ = 0.208, KS: *d* = 0.0616; *p* = 0.891) as illustrated on Figure 6C.

#### Standard Deviations (StD)

Standard deviations were calculated as they are used in clinical evaluations. The standard deviations of the reaction times (TRS, TR1C, and TR2C) of one subject reflected the dispersion of the subject’s responses. For RTT, mean = 50.918; median = 50.739; StD = 22.175. For 1CRRT, mean = 102.254; Median = 88.924; Std = 49.776. For 2CRRT mean = 109.635; median = 88.548; Std = 65.129.

### Changing the Measurement of the Perceptivo-Motor Reaction Time Part

Godefroy et al. (1999) suggested that a way of measuring the perceptual-motor reaction time is to measure the minimum response time rather than the average RT. We thus studied the distribution of minimum RRT and considered redefining our difficulty scale by subtracting the minimum RRT instead of the average RRT, from the average 1CRRT and 2CRRT before computing the coefficient that would align the means. Not surprisingly, these 2 RRT scores were correlated [Pearson corr (meanRRT, minimumRRT), *r* = 0.68, *p* = 6e-6]. We found that the difficulty coefficient was about 1.44 and that the distributions of (1CRRT-RRTmin) and (2CRRT-RRTmin)/1.44 were not significantly different (*p* = 0.71). The benefit of one method compared to the other is not clear, and the study should be pursued with larger numbers of subjects.

### Error Types

**FIGURE 7:**
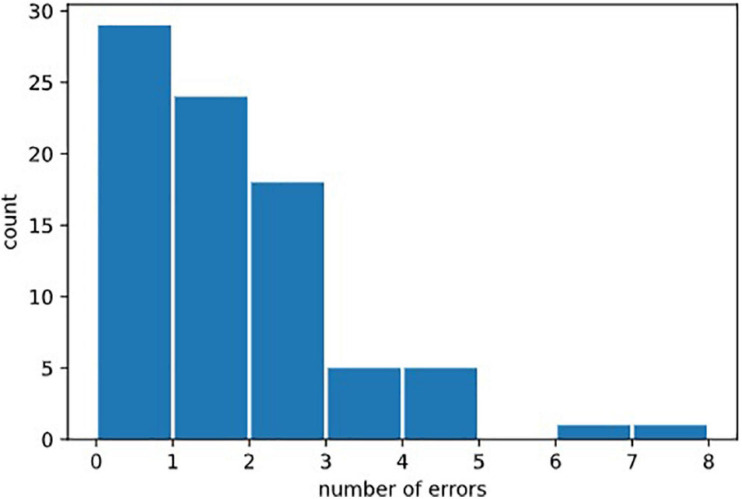
Distribution of erors. Statistical distribution of numbers of errors could not be fitted by a normal law, because it was bounded on the left by the minimal value 0, and by the fact that 0 was the most probable value (in healthy subjects). It would be natural to think that it could be fitted by exponential law (Poisson distribution) but the amount of errors in our data was not sufficient to draw this conclusion.

We defined several types of errors: anticipatory errors, omission errors and choice errors (Cf. Figure 2). However, as errors were rather rare in our healthy population, we calculated the total of all errors in the three test parts named “Errors.” Errors = Anticipatory error + Omission errors + Choice errors. Error mean = 1.31, Error median = 1; Error Std = 1.46. See Figure 7, for the statistical distribution of Errors. This distinction may prove to be interesting in clinical populations.

### Re-test

The re-test effect was observed in a subgroup of 28 subjects with the test conducted in the same conditions 24 h later.

## Results: Examples of Use and Limitations

### General Results

Our method is based on the notion of increasing the TR linked to the difficulty, allowing us to calculate a difference between the “choiceReactionTimes” and RRT (Tables 1, 2). 100% of the subjects in this study had a positive difference.

**TABLE 1:** Distribution of our sample.

	Total	Male	Female
*N*	83	20	63
Age	19–45 (29,3)	Avg. = 31.4, std = 9.7, median = 31.5	Avg = 28.7, std = 7.9, median = 26.0
Laterality	Left (13.25%)	3	8
	Right (86.75%)	17	55

**TABLE 2:** General results.

	Mean	Std	95% confidence interval
RRT	309.56	48.02	298.95−320.18
1CRRT	513.81	105.56	490.47−537.15
2CRTT	634.71	162.59	598.77−670.64
1CRRT′	204.24	93.45	183.59−224.90
2CRRT′	325.14	155.47	290.78−359.50
2CRTT′′	204.25	97.66	182.66−225.83
ES	204.24	83.89	185.70−222.79
Delta	0.00	45.80	−10.12−10.12
RD	0.01	0.20	−0.04−0.05

### Distributions

The distributions of reaction times, RRT, 1CRRT, 2CRRT, 1CRRT′, 2CRRT′′, and ES can be fitted by Gaussian Normal distributions. ES Gaussian fit (μ = 195.09, σ = 84.46, KS *d* = 0.081, *p* = 0.61). RRT Gaussian fit (μ = 305.802, σ = 51.228, KS *d* = 0.060, *p* = 0.90). However, we preferred to fit them by ex-Gaussian distributions, which are more commonly used in the field (Table 3).

**TABLE 3:** Ex-Gaussian fits of the distribution.

	Mu	Sigma	Tau	KS-test *p*
RRT	264.41	33.01	46.39	97.32%
1CRRT	400.22	57.25	119.23	91.13%
2CRTT	494.37	81.46	131.52	KS-test *p* = 96.68%
1CRRT′	121.48	66.88	82.71	KS-test *p* = 92.71%
2CRTT′	175.29	50.34	147.00	93.84%
2CRTT′′	110.12	31.62	92.34	93.84%
ES	mu = 124.25	53.51	80.13	96.94%
Fit Gaussian				
Delta	0.00	38.6		88.3%
RD	−0.01	0.21		89.1%

An even better fit would be with the diffusion model (Ratcliff and McKoon, 2008), but that depends on 7 parameters, and requires much more data that are often not accessible in practice.

### Correlations

Pearson’s correlations: We observed that RRT, 1CRRT, and 2CRRT were correlated, corr (RRT, 1CRRT *r* = 047, *p* = 9.1e-06); corr (RRT, 2CRRT *r* = 0.29, *p* = 0.0072); corr (1CRRT, 2CRRT *r* = 0.59, *p* = 3.1e-09). We recall that we acknowledged that they are composite, and that we can break them down into their elementary components, RRT, ES, and RD. This decomposition was confirmed by the fact that RRT, ES, and RD were indeed not correlated (*p* > 0.05).

ES was not correlated to RRT, but it was logically correlated with 1CRRT and 2CRRT, corr (ES, 1CRRT *r* = 0.77; *p* = 9.3e-18); corr (ES, 2CRRT *r* = 0.85, *p* = 7.1e-24).

The volunteers filled out the HADS anxiety and depression self-questionnaire (we recall that our criteria excluded diagnosed depression). Scoring for anxiety and depression was not correlated.

**FIGURE 8:**
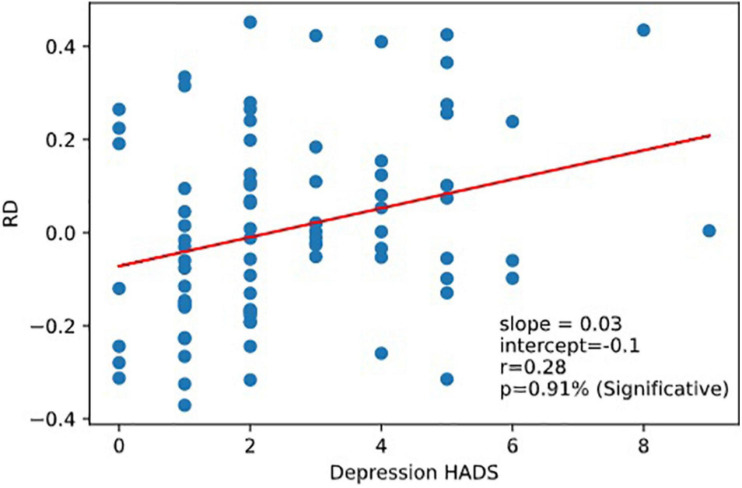
Correlation RD vs.depression. Reaction to dfficulty (RD) is correlated to depression score of the Hospital Anxiety and Depression Scale (HADS).

Very interestingly, we observed that RD was correlated to the Depression part of HADS responses corr (RD, Depression *r* = 0.28,*p* = 0.0091). RD was not correlated with the reaction time scores (nor with RRT, 1CRRT, 2CRRT, and ES) nor with Error (Figure 8).

**FIGURE 9:**
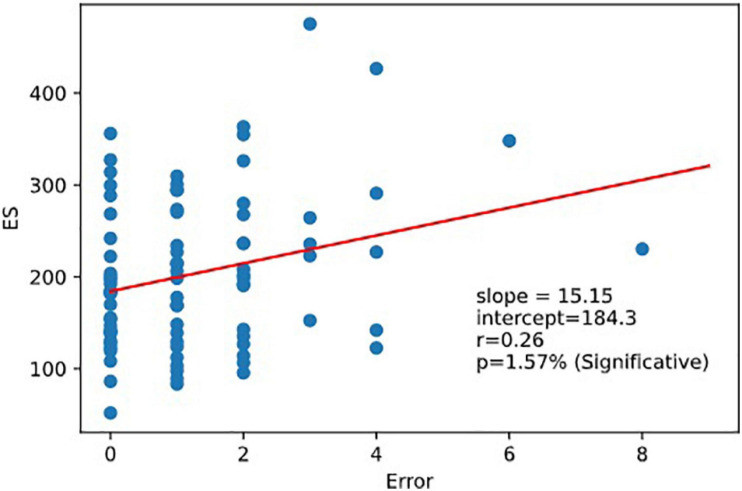
Regression ES vs.error. Executive speed and error are correlated. Linear relation between executive speed and errors. Pearson coefficient of regression (ES, Errors *r* = 0.264 *p* = 0.016).

As expected, ES was correlated to 1CRRT and 2CRRT, corr. (ES, 1CRRT) *r* = 0.77, *p* = 9.3e-18; corr (ES, 2CRRT) *r* = 0.85, *p* = 7.1e-24 and ES was not correlated to RD nor to the HADS and its 2 subscores, depression and anxiety. However, we observed that there was a linear correlation between ES and Errors (the number of errors) (Figure 9). There was not a linear regression between Errors and RRT (Pearson: *r* = −0.174, *p* = 0.12).

No correlation with age was found in this study, but this could be due to the homogeneous age range of our sample and would need to be investigated further.

### Test Consistency and Reliability

#### High Level of Internal Consistency

##### Convergent and divergent internal correlation method

We observed very strong internal consistency (across items) assessed by the convergent and divergent internal correlation method. Our model assumes that RRT, 1CRRT, and 2CRRT all contained a “RRT part.” Thus, RRT was, as expected, strongly correlated with 1CRRT and 2CRRT, Pearson corr (RRT, 1CRRT *r* = 047, *p* = 9.1e-06); corr (RRT, 2CRRT *r* = 0.29, *p* = 0.0072); corr (1CRRT,2CRRT *r* = 0.59, *p* = 3.1e-09).

Divergent internal correlations: separation of RRT and ES was confirmed while RRT and ES were not correlated with each other. Separation of the RD coefficient was confirmed while RD was not correlated to RRT nor to ES.

##### Validity of construct

RRT, 1CRRT, and 2CRRT had different means, confirming the pattern of the increase in reaction time with increasing difficulty of the response. KW test (RRT, 1CRRT, 2CRRT, *s* = 171.32, *p* = 0.00627).

##### Single-administration test score reliability: Cronbach’s alpha

Single-administration test score reliability – Cronbach’s alpha (Tau-equivalent reliability) are all at very good levels of reliability > 0.9. RRT: Cronbach Alpha RRT = 0.9147; 1CRRT = 0.9083; 2CRRT = 0.9551.

#### Reliability (Test and Re-test Reliability)

We tested the evolution between day zero and day 1 (the following day) in a subgroup of 28 subjects.

**FIGURE 10:**
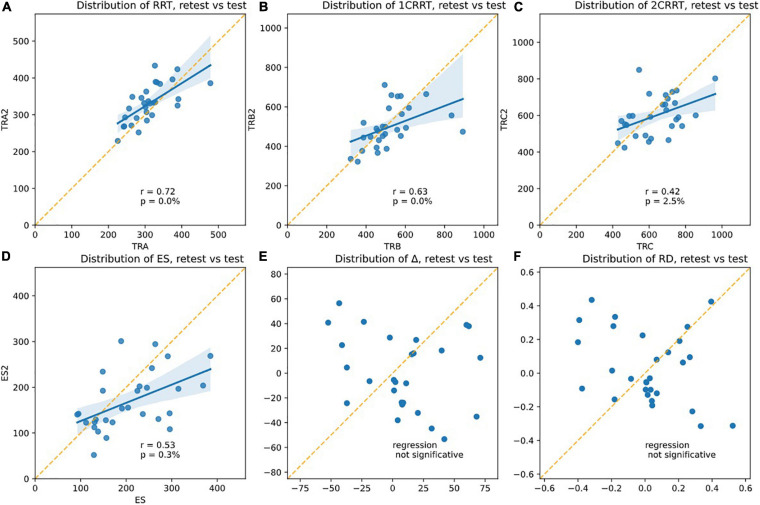
Plotsshowing the test-retest effects. The slope of the linear regression asserts the Pearson *r*-coefficient. The correlation is significative for **(A)** RRT, **(B)** 1CRRT, **(C)** 2CRRT, **(D)** ES, and not for **(E)** Delta and **(F)** RD. An ideal test would have *r = 1* (regression line parallel to the diagonal), but this never happens in any reaction time tests.

Re-test reliability can mean that the measurements would be identical the second time (assessed by a paired comparison of means). However, this almost never occurred in the RT tests found in the literature: all have a retest effect usually described as a notion of “novelty.” Another assessment of reliability is whether the values the second time are correlated to the values the first time. From our data (see Figure 10), we found that Test and next-day-Retest reliability was good for RRT, Spearman correlation of ranks are: corr(RRT0,RRT1, *r* = 0.72, *p* = 0.00%) Figure 10A; corr(1CRRT0,1CRRT1, *r* = 0.63, *p* = 0.02%) Figure 10B;corr(2CRRT0,2CRRT1, *r* = 0.42, *p* = 2.51%) Figure 10C; corr(ES0,ES1, *r* = 0.53, *p* = 0.31%) Figure 10D; corr(Error0,Error1, *r* = 0.13, *p* = 51.68%).

Most RT tests in the literature have r in the range 0.5–0.8.

Correlation for number of errors was not significant, but this could simply be related to the fact that few errors were produced by our healthy subjects.

Yet another assessment of test-retest reliability is by ANOVA Intra Class Correlation (ICC), by taking the ratio of intra-class variance to total variance. *r* = 1 would mean perfect reliability, and *r* = 0 would mean no reliability. Most RT tests in the literature have r in the range 0.5–0.8.

ICC ANOVA: percentage of the total variance due to IntraClass (i.e., not from test/retest Inter-Classes): RRT: ICC = 98.5%, 1CRRT ICC = 98.8%, 2CRRT ICC = 97%.

Very interestingly, Δ (Figure 10E) and the difficulty reaction score was not correlated on re-test: corr(RD0,RD1, *r* = −0.30, *p* = 10.83%); (Figure 10F). The subjects did not react in the same way to the difficulty.

**FIGURE 11:**
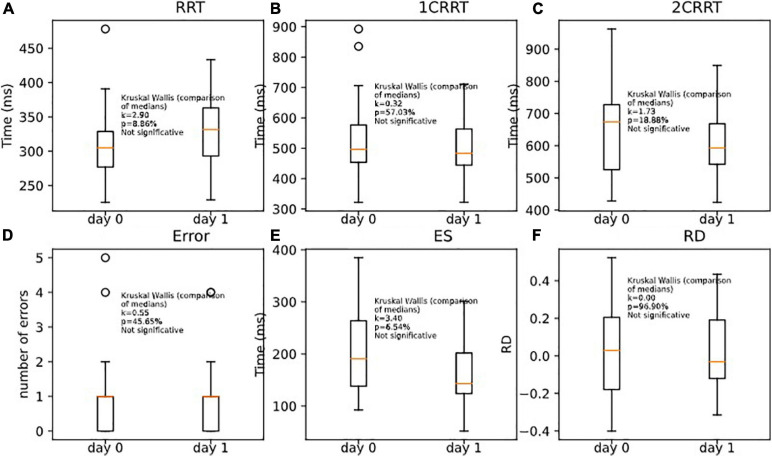
Comparison of medians for re-tests effects on **(A)** RRT, **(B)** 1CRRT, **(C)** 2CRRT, **(D)** Errors, **(E)** ES, and **(F)** RD. Kruskal-Wallis test asserts that medians are not significantly different.

Unsurprisingly, the subjects remained in the same areas of TR (the mean of T + 1 days is in the area of the standard error of the first pass). Nonetheless, we observed a tendency to go a little faster in the most demanding tasks (with categorization). This effect was only observable on the executive time part (ES) and not in the perceptivo-motor time part (RRT). As RRT is contained in 1CRRT and 2CRRT, the effect was not observable, but it appeared specifically when we subtracted the RRT part, in the ES. Kruskal–Wallis (RRT0,RRT1, *k* = 2.90, *p* = 8.86%) (Figure 11A); (1CRRT0,1CRRT1, *k* = 0.32, *p* = 57.03%) (Figure 11B); (2CRRT0,2CRRT1, *k* = 1.73, *p* = 18.88%) (Figure 11C); (Error0,Error1, *k* = 0.55, *p* = 45.65%) (Figure 11D); (ES0,ES1, *k* = 3.40, *p* = 6.54%) (Figure 11E), (RD0,RD1, *k* = 0.00, *p* = 96.90%) (Figure 11F); this was a very interesting result which suggests that the retest effect was linked to the decision time (reflecting executive process), and not to the perceptual-motor time.

**FIGURE 12:**
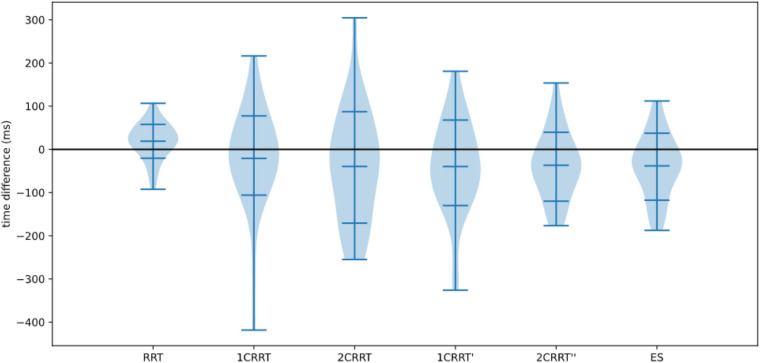
Distributions of differences re-test–test.

In Figure 11 we plotted the distributions of reaction times for test and retest, and in Figure 12 we plotted the distribution of the difference.

#### Consideration About Alternative Methods

Testing the methods of Godefroy et al. (1999), i.e., taking as a baseline (that we subtracted from the choice reaction times) the minimum RRT = minRRT, instead of mean RRT, we looked for the method that would provide the best test–retest stability for an individual. We tested Pearson correlations of min day0 and min day1 and found: corr (minRRT0, minRRT1, *r* = 0.51, *p* = 0.0014); corr (min1CRRT0, min1CRRT1, *r* = 0.47, *p* = 0.0037), corr (min2CRRT0, min 2CRRT1, *r* = 0.7, *p* = 2.5e-06). This method showed correct test-retest stability but was not better than our method (by subtracting the average from the RRT). We therefore kept the method of removing the mean or the median rather than the minimum RRT obtained. It will be interesting to pursue investigation on these questions in the future. It is noticeable, however, that removing the average removed some of the attentional functions contained in the variability of the RRT. Since we subtracted the RRT from the go/no-go tasks RT, this did not affect the measures of ES or RD.

## Discussion

### Objectives

Our goal was to present our technique to dissociate RTs in order to provide clinicians around the world with a short digital test to assess decision-making skills and disabilities by taking into account the particularities of the subject’s reaction following his/her own perception of the complexity of the task. We hypothesized that depending on their capacities of adaptation, individuals will play on both speed and precision, with some subjects going faster while others slow down when facing difficulty. Finally, the ultimate goal was to observe the balance governing the speed-precision compromise that each individual achieves.

We can summarize our method as follows: By taking three reference points requiring attentional and executive processing of different complexity we introduced a method for comparing the reaction times of different tasks, making it possible to calculate the deceleration specific to the reaction time linked to complexity. On a set of healthy subjects, we defined the difficulty coefficient of a task as being the ratio of the average time of this task minus the base time/average time of the unit task minus the base time. As we know that a subject’s motor response undergoes large individual variations (Gueugneau et al., 2017), it seemed important to distinguish the component linked to attentional and executive processes from that associated to perceptual-motor latency. We found that RT was composed of 3 more elementary uncorrelated components. We hypothesized that the first part RRT, not linked to the difficulty of the task, reflects the perceptivo-motor component, that ES reflects the ES, and RD reflects how the subject reacts to difficulty by accelerating (RD < 0) or decelerating (RD > 0). Recording the number of errors and their type also gives a picture of precision of the subject’s response. The objective was to capture the balance between speed and precision in perceptual-motor decision-making. Ecologically, it is an essential issue as it allows adaptation to a potentially changing environment (Godefroy et al., 2018). In addition, it is a unique index of a subject’s reaction to the perception of the difficulty of a given task.

### Two New Indices of Brain Function

#### Executive Speed

The need to process reaction time to extract executive components from perceptual-motor components comes from the known between-subject and within-subject variability in psychomotor speed. This variability is linked to a natural variation of the perceptual-motor part of processing speed in aging (Ebaid et al., 2017) and because execution parameters, motor planning mechanisms are modulated during the day (Moore, 1997; Gueugneau et al., 2017). Since between-subjects perceptual-motor times are less fluctuating than decision time, reaction time is more the reflection of the time needed to decide (Noorani, 2014). As a consequence, RTs measurements need to separate perceptual-motor and execution time, to better understand the origin of the variations in a given subject, in particular if a pathology is suspected.

Our model takes this variation into account by providing for each subject his/her own initial baseline regarding perceptual-motor time. However, calculating a simple ratio, as is the case for Trail Making Tests [i.e., an index of executive function is provided by the B/A ratio (Arbuthnott and Frank, 2000)], assumes that there is a proportionality between the executive time and the perceptual-motor time. In our paradigm, we wanted to observe executive time by clearly separating it from perceptual-motor time. Moreover, if we want to go further in the observation of the fundamental elements of executive functioning, it is necessary to be able to acquire more data and, in particular, to have at least three levels of RT measurements, all of them with comparable movement time. Calculating the second derivative makes it possible to extract from these 3-reaction time measures the Reaction to Difficulty, which is a new measure of brain function.

Our approach was a little different from that of Godefroy et al. (2010), while adopting the same reasoning of the separation of the perceptual-motor part of the slowing from an attentional part. Godefroy and his team argued that the time of action depends heavily on motor and perceptual processes; attention is considered to be an extension of the peak and right tail of the RT distribution. They assessed the “generalized slowing” (the extra time taken to perform a dual-task compared to a single reaction time task) by taking the ratio of the dual-taskRT, which amounts to taking the single taskRT as a baseline. In our study we also consider the RRT as the baseline perceptual-motor part and we take the executive part as the difference with the baseline rather than the ratio. Taking the difference rather than the ratio has the advantage of producing an index that is decorrelated from the simple RT, whereas the ratio remains correlated to simple RT.

Our study is compatible with the diffusion model (Ratcliff and McKoon, 2008) (a fundamental model for describing reaction times). One of the parameters of the diffusion model amounts to subtracting a baseline time (the non-decision-time) to RT and considers that the remaining part is proportional to a drift parameter linked to the difficulty of the task (its perception by the subject). This is precisely what we do in our approach and confirm in our data.

The only difference between the 1-Choice-reaction time and the 2-Choice-reaction time is the difficulty of the task, and we find that the RT distributions are proportional. This is what is predicted by the diffusion model if we assume that the only parameter that changes between the 2 tasks is the drift parameter. Our approach, consisting in increasing the difficulty of the task while keeping everything else constant, can be viewed as a way of measuring the drift parameter of the subject. Our coefficient of difficulty can be interpreted as the measure of the drift parameter of the 2-Choice-task, in units of the 1-Choice-task. It seems important to explore this idea further in larger studies.

Other important parameters to take into account are the temporal course of the signal and the subject’s inhibitory abilities. Inhibition is a fundamental cognitive function involved in every organized cognitive behavior (Bari and Robbins, 2013). A model-based approach (Mulder and Van Maanen, 2013) provides a subtle explanation of how the underlying dynamics of the decision process might give rise to these various effects. The validity of the attentional cue affects the decision-making process, but the temporal proximity of a cue might interfere with general processing of a subsequently presented target stimulus (Mulder and Van Maanen, 2013). Therefore, in the speed/accuracy compromise, the subject’s inhibitory abilities come into play. Choice RT paradigms integrate information in a gradual process with at least one stimulus-related level and one response-related level made of information accumulators: a given response is emitted as soon as one accumulator reaches a predefined threshold. Therefore, RT is a function of the “time” necessary to reach this threshold. When only one response is correct, the possible responses are in competition to reach the threshold first. Burle et al. (2004) demonstrated that the activation of the motor structures involved in a required motor response is accompanied by the inhibition of structures involved in alternative responses and that inhibiting incorrect responses shares some mechanisms with the need to withhold a response.

#### Reaction to Difficulty Quantification

Our method allowed us to define two new indices of brain functions: ES (which is the executive time dedicated to selecting the correct answer) and Reaction to Difficulty.

We show here that Reaction to Difficulty (RD) is correlated with the Hospital Anxiety and Depression scale, HAD (Zigmond and Snaith, 1983), and more precisely with its “Depression” component but not with its “Anxiety” component. This result suggests a relationship between our new index of brain function, Reaction to Difficulty, and the subject’s emotional state.

Very interestingly, while the test and retest consistency of RTs was excellent, the RD score was not correlated between test and retest. This observation reinforces the hypothesis of a link between RD and the subject’s emotional state. Indeed, subjects may react differently on the second day as compared to the first day. Some participants reacted by taking a little less time in the most complex part of the test whereas others took a little more time. This suggests that the former benefited from a learning process (and felt more confident) while the latter anticipated difficulty (and became more cautious). This result is consistent with studies showing a link between emotion and reaction time. Emotions modify response times in the initiation of motor actions and emotional states influence the speed with which goal directed movements are initiated (Beatty et al., 2016).

We previously showed that in healthy subjects there were specific and different patterns of activation associated with different levels of performance in a decision-making and planning task (Cazalis et al., 2003). These data evidenced that, beside behavioral outcome, healthy subjects react to task difficulty by mobilizing distinct neural networks. In this previous work there were higher and lower performers who mobilized distinct neural networks. In the work presented here, we raised the question of similar or different neural networks that a person mobilizes according to his/her way of coping with higher levels of difficulty.

Our most fundamental result is the observation that the measures of cognitive processing of single categorization on the one hand and double categorization on the other hand show a similar statistical distribution, after rescaling double categorization via a coefficient of difficulty. This raises many questions about brain functions and cognitive processes and will require specific research in the future. We hypothesize that the 2 similar distributions [1CRRT-RRT and (2CRRT-RRT)/Coef], with ES being the average of the 2, are undertaken by the same neural networks and that the reaction to the difficulty is supported by another network, in interaction with the first one.

The processing of speed and response variability is very well illustrated in ADHD, for which it is one of the best predictors of emotional lability (Kuntsi et al., 2010). Indeed, the link between emotional lability and neuropsychological variables (most strongly by processing speed and response variability) in ADHD patients cannot be explained by cognitive or motivational deficits (Banaschewski et al., 2012). Therefore, by breaking down the speed process (i.e., psychomotor slowing) into elementary processes (perceptual-motor reaction time, ES, and Reaction to Difficulty, which is itself correlated to emotional processes as seen in the depressive questionnaire), the MindPulse test could be expected to provide meaningful information for this pathology.

### The Balance Between the Response Speed and Its Accuracy

We observed that there is a linear relation between ES and Errors, thus providing support for the idea that selective attention is the result of a balance between speed and precision which may be continuously readjusted according to experience. The fact that Errors are correlated to ES confirms this interpretation. On the other hand, we observed no relationship between the RRT and Errors, thus reinforcing the idea that the attentional component of the task is indeed contained in ES and mostly excluded from RRT. This very important result will have to be reproduced for confirmation and/or completed with a larger number of subjects. Another question that it will obviously be important to raise is how the speed-precision balance breaks down for each type of error. In particular, it would be important to establish whether the same mechanism applies for anticipation, omission, or choice errors.

In our test, we can observe the average balance reached by a person, taking into account his/her speed, precision, errors (which influence an adjustment of the subject) and his/her reaction to difficulty. The notion of reaction to difficulty raises the question of whether it refers to the system of permanent adjustment to errors described by Vidal et al. (2020), or if it is an emotional reaction, or finally if the two phenomena work together.

### Validity, Scalability, and Limitations

We showed the validity of our model and the ability to extract new markers of healthy brain function by analyzing reaction times with at least three conditions of different complexity. Consistency and reliability were very strong and showed the good validity of our construction. We did not evaluate consistency across different laboratories (inter-rater reliability) which makes little sense in this study, since examiners have only a minor role and take no measurements. With written instructions, subjects without cognitive impairment can even take the test on their own. In subsequent studies, however, it will be interesting to observe the consistency of the measurements on different operating systems (Mac versus Windows and their various versions) and the inter-rater reliability of examinations with patients needing assistance.

As the type of stimuli, the way in which they are presented, and the motor response are strictly identical from one subject to another, our analyses provide a reliable index revealing how a subject reacts to increasing decision difficulty, and to simultaneously observe the speed/accuracy compromise.

The test was constructed in such a way as to avoid the retest effect as far as possible, but we know from the literature that there is an effect of pre-exposure on executive functioning score in general (Calamia et al., 2013) and in reaction time tests (Lowe and Rabbitt, 1998). The “waiting time” (time elapsing before the stimuli appeared on the screen), and the categories to respond to were randomly attributed between subjects. Correct and incorrect responses were equiprobable in the two choice tasks, making it impossible for subjects to predict the response. In the subgroup who took the test again the following day we did not observe any retest effect for RRT, but we observed a tendency to go a little faster on the executive time part (ES). This result supports the idea that the ES measures something different from the RRT. However, it is clear that the retest effect will need to be evaluated on a sample of people representative of the population and by observing the variations of this effect from 1 day to 6 months.

Variance is inherent in these tests and lengthening the repetitions and the duration of the experiment tends to increase the variance rather than stabilize it. It is therefore extremely difficult to measure reaction times with a higher precision. However, the variance is an interesting index in itself, as argued by several articles. The issue is not really the variance but rather the presence in the data of very slow responses (the extremes). A meta-analysis investigating the RT variability in controls and ADHD children, adolescents, and adults (Kofler et al., 2013) concluded that the variability in the task performance of ADHD was primarily due to a set of abnormally slow responses, rather than ubiquitous variability across all trials in the task. In similar tasks to those of the MindPulse test, children with ADHD showed increased variability on simple and complex Go/No-go tasks (each task lasting round 8 min) with significantly increased RTs on the complex Go/No-go task. Given that children with ADHD show higher variability, the slower RTs on the complex task may not reflect a generalized pattern of slowing but rather, occasional “outlier”slow responses (Vaurio et al., 2009). Variance also concerns pathologies such as schizophrenia where increased reaction time intra-subject variability in fast decision tasks has been confirmed in patients and might be linked to a deficit in the inhibitory control of action (Panagiotaropoulou et al., 2019). From the analysis according to the three parameters of ex-Gaussians, our data are generally comparable with those in the literature (Swick et al., 2013; Dotare et al., 2020). Our values of tau are slightly higher, which shows that our test tends to generate extreme RTs, which is recognized as being a good indicator in certain pathologies of attention such as ADHD (Tarantino et al., 2013).

Our objective is to seek for an overall equilibrium, in a relatively short test with an approach consisting in first removing a “perceptual-motor” part from the data, before breaking down the residual RT.

The analysis of the shape of the RTs curve, as in ex-Gaussian (Tarantino et al., 2013), which generally requires a lot of trials over a long period of testing, can be considered, even if it is not our main objective, we show in this article that our data allow us to obtain these curves. However, the interest and the ability to do so with the test will have to be demonstrated in pathological conditions.

We do not seek to measure reaction times over a long period of time but to use precise reaction time measurements to look at aspects of fundamental decision-making, by superimposing tests to derive more basic components, the objective being to finally having clues which can then separate the normal conditions from the pathological ones with a few set of data. In this we are closer to the Stroop (Stroop, 1935) or Trail Making Test (Reitan, 1992). These tests are largely useful in clinical neuropsychology with very few data taken: For example, The TMT takes 2 times measurements and 2 speed measurements, the Stroop, depending on the version, between 3- and 4-timesmeasurements and 3 or 4 precision measurements. By taking more data, with a test that remains in the category of “short tests,” we wanted to repeat the exploit of these known tests, to provide useful indices while remaining very short and taking little data.

The test has an excellent potential for scalability, as it is a computer software that can be downloaded online and installed on most computer systems.

As this first study was just a proof of concept, our population is not representative of the general population, with a very homogeneous group in terms of age and socio-cultural level, and a large majority of women. These results will have to be reproduced on a more varied and larger population.

The reaction to difficulty could also be related to the residual fluctuation of the data, but the fact that the data show a correlation (*p* < 1%) with the HADS depression score, encouraged us to propose that RD contains an emotional component. Subsequent studies should take up this idea, on the one hand by analyzing this phenomenon on a large, socially representative sample of people, and on the other hand with clinical studies on subjects having an emotional state that alters their ability to make precise and fast decisions (for example, depression).

The test shows a great sensitivity to measures of RT. It is therefore necessary to scrutinize the extreme responses very carefully, for example the extremely slow responses which can be caused by external disturbances.

### Practical Implications and Suggestions for Future Research

We provide here a first proof of concept of our ability to measure 4 axes of the speed-precision balance of a subject’s fundamental decision making: these axes are perceptual-motor speed, ES, subject accuracy and reaction to difficulty.

We plan to continue our research on the one hand on the possibility of using MindPulse as a tool for the clinical evaluation of cognitive functions and on the other hand on the calculation of the difficulty gradient.

With the objective of using MindPulse as a clinical test, our goal now will be to repeat these results on a larger and less homogeneous population by performing a MindPulse assay calibration (normative data on a large and representative population). The initial results presented here have raised many questions regarding its utility as a test for cognitive functions: issues that require further exploration concern from what age it can be used, how to handle aberrant responses according to their distribution, and how to evaluate the contributions of the different types of errors to the analysis. We also need to launch clinical trials in several pathologies known to involve problems of psychomotor speed and attentional functions in order to assess both sensitivity and specificity.

From a fundamental research point of view, other questions are interesting: to measure the speed of cognition and perception of stimuli, the order of appearance of the conditions of “difficulty” could be reversed; a fourth level of difficulty could also be added to calculate the consistency of the calculation of the difficulty index, and it would also be interesting to investigate whether this model is linked only to the visual modality, or if the same kind of results are found with the auditory modality for example.

We propose a general method to compare tasks of the same kind but of different difficulty in general. Our method consists in first devising at least three tasks that are strictly comparable in stimuli presentation and motor output. One of the tasks must be the base, its characteristic being to be sufficiently basic so that its level of difficulty is as low as possible (level “zero” difficulty). We then define another task as the one that will serve as a reference for the unit of difficulty (the task of “difficulty 1”). Our method is to calculate the “difficulty coefficient” to make other tasks comparable to our level zero and one. We speculate that whatever new task we introduce will always have the same statistical distribution for ES. This rescaled variable appears to be a fundamental signature of attentional functioning.

## Data Availability Statement

The original contributions generated for this study are included in the article/Supplementary Material, further inquiries can be directed to the corresponding author.

## Ethics Statement

Ethical review and approval was not required for the study on human participants in accordance with the local legislation and institutional requirements. The patients/participants provided their written informed consent to participate in this study.

## Author Contributions

All authors wrote the manuscript and agreed to be accountable for the content of the work. SS and SG obtained the data. BE wrote the software. SS and BE analyzed the data.

## Conflict of Interest

All authors declare that they are listed as inventors of the MindPulse test with a patent pending. SS declares that she is President and Researcher at It’s Brain (Orsay, France), the company that is currently developing the MindPulse. The studies and developments presented in this article were carried out before the creation of It’s Brain company. None of the authors were paid by a company for this study.

## Abbreviations

1CRRT: 1-choice-releasing reaction time
2CRRT: 2-choice-releasing reaction time
ES: executive speed
RD: reaction to difficulty
Errors: total errors.
